# 

**Published:** 1890-10

**Authors:** 


### CONTENTS.

### COMMUNICATIONS.

Operative Dentistry.......... 473
Anæsthesia.......... 479

### PROCEEDINGS.

The Thirtieth Annual Meeting of the American Dental Association 481

### SELECTIONS.

Thymol as an Intestinal Antiseptic.......... 510
The Relation of Mastication to Physical Development.......... 510
The Wearing Away of Teeth.......... 512
Experimental Investigations Concerning Suppuration.......... 513
Dentition.......... 514
Nerve Restoration.......... 516
Disinfecting Power of Peroxide of Hydrogen.......... 517
The International Congress from an English Standpoint.......... 518
Anæsthesia—Nitrous Oxide.......... 519

### EDITORIAL.

American Dental Association.......... 520
Change of Time.......... 521
Ohio State Dental Society.......... 521
Bibliographical.......... 522
Contagion Vivum.......... 523
Obituary.......... 524

